# The Impact of Perioperative Radiotherapy on Disease-Specific Survival in Patients with Localized Retroperitoneal Liposarcoma: A Population-Based Propensity-Score Matched Analysis

**DOI:** 10.1245/s10434-024-16703-w

**Published:** 2024-12-16

**Authors:** Alexander Wilhelm, Benjamin Wiesler, Christoph Kümmerli, Markus W. Gross, Christoph Kettelhack, Beat P. Müller

**Affiliations:** 1 Department of Surgery, Clarunis – St. Clara Hospital and University Hospital Basel, Basel, Switzerland; 2 Surgical Outcome Research Center Basel, University Hospital Basel, University Basel, Basel, Switzerland; 3 Department of Radiooncology, University Hospital Basel, Basel, Switzerland

**Keywords:** Retroperitoneal liposarcoma, Retroperitoneal sarcoma, Radiotherapy, Disease-specific survival, Overall survival, Propensity score matching, Population-based study, SEER

## Abstract

**Background:**

The impact of radiotherapy on the oncologic outcome of retroperitoneal liposarcoma (RPLS) remains controversial. The aim of this study was to evaluate the effect of radiotherapy on disease-specific survival (DSS) in a cohort of patients with RPLSs.

**Methods:**

In this population-based, retrospective cohort study, patients with localized RPLSs who underwent surgical therapy were identified from the Surveillance, Epidemiology, and End Results-17 cancer registry program. After propensity-score matching for potential confounders, multivariable logistic and Cox regression analyses were used to examine factors associated with DSS and radiotherapy.

**Results:**

From 2004 to 2020, 1692 patients with localized RPLS who underwent surgical therapy were identified (84.2%  White, 44.6% female, mean age 62 years). Of those patients, 393 patients (23.2%) received perioperative radiotherapy. Patients who received radiotherapy had a higher rate of tumor size between 10 and 20 cm and unknown tumor grading. After propensity-score matching, multivariable adjusted Cox regression and Kaplan–Meier survival analysis demonstrated no improvement of DSS for patients who underwent radiotherapy (hazard ratio 1.04, confidence interval 0.81–1.32; log-rank *p* = 0.47). Patient age ≥80 years, larger tumor size, and tumor grading G3 versus G1/2 were associated with an increased risk of death due to RPLS. Subgroup analyses stratified by grading showed similar outcomes.

**Conclusions:**

The administration of perioperative radiotherapy did not improve DSS in patients undergoing surgery for localized RPLS in this population-based study. Therefore, the use of perioperative radiotherapy in these patients may be questioned. However, the findings should be interpreted with caution due to the inherent limitations of the Surveillance, Epidemiology, and End Results (SEER) database.

**Supplementary Information:**

The online version contains supplementary material available at 10.1245/s10434-024-16703-w.

Retroperitoneal sarcoma (RPS) is a rare disease, with an incidence of two to three new cases per 100,000 people per year, representing 1% of all solid malignancies.^1^ The most common histological subtype is well and dedifferentiated liposarcoma (65%), followed by leiomyosarcoma (25%).^2^ RPSs are usually treated in highly specialized centers in multidisciplinary teams, with en bloc resection being the primary treatment.^3–5^ The rate of local recurrence remains high at up to 50%, even in these highly specialized centers.^6,7^ Therefore, significant importance is placed on neoadjuvant and adjuvant therapies.

The currently available evidence regarding the effect of perioperative radiotherapy on oncological outcomes in RPS is conflicting. Two population-based retrospective studies have shown an improvement in survival in heterogeneous groups of patients with localized RPS who underwent surgery with perioperative radiotherapy compared with surgery alone;^2,7^ however, the international trend is towards a decrease in the number of postoperative radiotherapy procedures. The majority of international guidelines advocate for preoperative radiotherapy over postoperative radiotherapy, primarily due to the higher toxicity profile, larger radiation target volumes, and increased incidence of adverse events following postoperative radiotherapy.^3,5,7–9^ In contrast, the prospective, randomized STRASS trial did not demonstrate a beneficial effect of preoperative radiotherapy on 3-year abdominal recurrence-free survival (RFS).^6^ Nevertheless, an exploratory subgroup analysis indicated the possibility of a protective effect in retroperitoneal liposarcoma (RPLS), which was subsequently validated in a pooled analysis of STRASS and off-trial (STREXIT) data.^10^

However, recruitment for the STRASS trial was challenging due to the limited number of RPS patients, and subgroup analysis has only exploratory value. Given the scarcity of RPS, a register-based analysis of a large cohort is necessary to confirm these previous findings. The STRASS trial examined abdominal RFS. It is unclear whether the positive effect of preoperative radiotherapy on the abdominal recurrence rate in localized RPLSs can also improve disease-specific survival (DSS). A recently published register-based study utilizing the National Cancer Database (NCDB) demonstrated that external beam radiotherapy did not result in a favorable impact on overall survival in RPLS.^11^ In comparison with overall survival, DSS provides a more focused perspective on the impact of a specific disease and the success of interventions for the treatment of that disease. The objective of this study was to perform an updated analysis of the Surveillance, Epidemiology, and End Results (SEER) database in order to determine the impact of perioperative radiotherapy on DSS in patients with localized RPLS.

## METHODS

### Data Source

This population-based study included patients with primary localized RPLS who underwent surgery between 2004 and 2020. Patients were identified from the SEER-17 cancer registry program of the National Cancer Institute.^12^ The SEER-17 dataset comprises data from 17 cancer registries in 13 US states, covering 26.5% of the US population. The last day of follow-up was 31 December 2020.

### Tumor and Treatment Characteristics

RPLS patients were identified using the International Classification of Diseases for Oncology Third Revision (ICD-O-3).^13^ Patients with code C48.0 for retroperitoneal primary sites were included. The study focused on the following histological subtypes of liposarcoma: liposarcoma not otherwise specified (8850), well-differentiated liposarcoma (8851), myxoid liposarcoma (8852), round cell liposarcoma (8853), pleomorphic liposarcoma (8854), mixed liposarcoma (8855), and dedifferentiated liposarcoma (8858).

The SEER database has included information on tumor size since 1983, with various changes over time. For the study period, patients who underwent surgery between 2004 and 2015 were categorized according to the Collaborative Staging Codes, and patients who underwent surgery between 2016 and 2020 were categorized according to the Tumor Size Summary codes. The categorization of tumor grading has undergone a number of modifications and adaptations. The ICD-O-3 classification was used to determine tumor grading for cases prior to 2018.^13^ In accordance with this classification, grading was subdivided into four categories: well-differentiated, moderately differentiated, poorly differentiated, and undifferentiated. After 2018, pathologic grade was recoded according to the American Joint Committee on Cancer (AJCC) staging system 7th edition,^14^ In the AJCC classification, tumors are graded as G1, G2, G3, and G4. These categories are equivalent to the ICD-O classification, thereby ensuring consistency over time.

The chemotherapy recode was used to determine whether patients underwent additional perioperative chemotherapy. Patients with unknown status were classified as not having received chemotherapy. Patients were subdivided into two groups according to their radiotherapy status using the radiation recode. Patients who received any perioperative radiotherapy (beam radiotherapy, combination of beam with implants or isotopes, radiotherapy not otherwise specified, radioactive implants, radioisotopes) were stratified into the ‘radiotherapy’ group, whereas patients who did not receive radiotherapy or who had an unknown radiotherapy status were stratified into the ‘no/unknown’ group.

### Statistical Analysis

Demographic, clinical, and pathological characteristics of patients undergoing surgery for RPLS between 2004 and 2020 and who underwent perioperative radiotherapy were compared with those who were not treated with radiotherapy using Student’s t-test for continuous variables and Chi-square tests for categorical variables. Patients who underwent radiotherapy were matched to patients who did not using nearest-neighbor propensity-score matching. A maximum of two patients who did not have radiotherapy were matched with each patient who underwent radiotherapy using a caliper of 0.1. Weighted matched standardized differences and variance ratios for the propensity-score model covariates were used to assess sample balance after matching. Acceptable balance was defined by a maximum of 0.1 for the standardized mean difference. The ‘optmatch’ and ‘Matchit’ package in R was used to perform propensity score matching (RStudio version 2022.02.3; www.r-project.org).^15–18^ The following confounding variables were included in propensity-score matching: age, sex, tumor size, and tumor grade. DSS was estimated using Kaplan–Meier analysis for patients with and without radiotherapy, and unadjusted comparisons between survival curves were made using the log-rank test. Multivariable logistic and Cox regression models were used to evaluate the associations of specific demographic and clinical factors with radiotherapy and the risk of death due to RPLS, respectively. Covariables were chosen based on clinical judgment and included: sex, patient age, tumor size, tumor grade, marital status, race, and systemic therapy. Patients with missing information on covariate data were excluded from analysis. All statistical tests were performed using a two-sided approach. A significance level of <0.05 was considered statistically significant. Statistical analysis was performed using Stata/BC version 16.1 (StataCorp LLC, College Station, TX, USA).

## RESULTS

### Demographic, Clinical, and Pathologic Characteristics

**Fig. 1:**
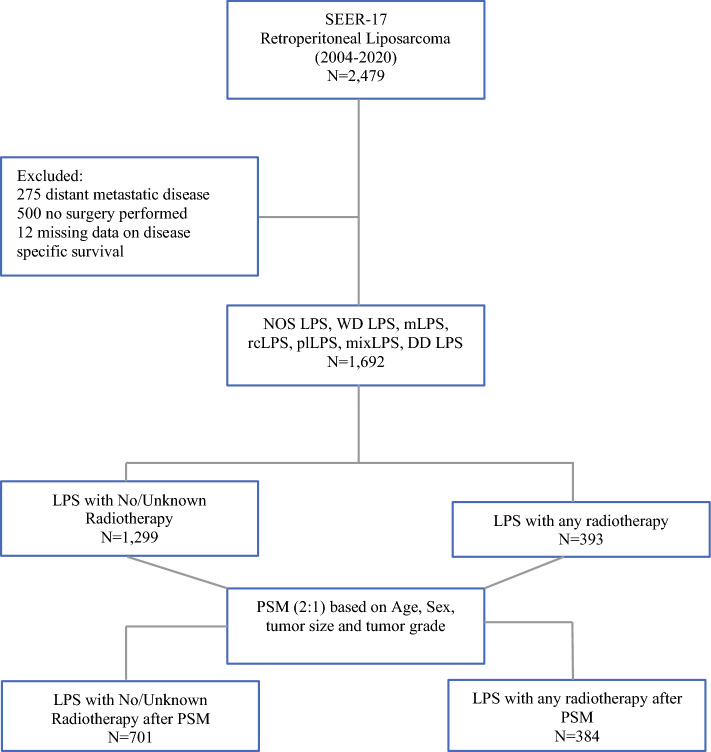
Participant flow diagram. *LPS* liposarcoma, *NOS LPS* liposarcoma not otherwise specified, *WD LPS* well-differentiated liposarcoma, *mLPS* myxoid liposarcoma, *rcLPS* round cell liposarcoma, *plLPS* pleomorphic liposarcoma, *mixLPS* mixed liposarcoma, *DD LPS* dedifferentiated liposarcoma, *PSM* propensity score matching, *SEER* Surveillance, Epidemiology, and End Results

From 2004 to 2020, a total of 1692 patients with localized RPLS who underwent surgery were identified in the SEER database (Fig. 1). Overall, the median age was 62 years, 84.2% of patients were White, and 44.6% were female. Of all patients, 1299 had a ‘none/unknown’ radiotherapy status (76.8%) and 393 patients received perioperative radiotherapy (23.2%). Of those patients who received radiotherapy, 35.3% (*n* = 139) underwent neoadjuvant radiotherapy. The most frequently employed form of therapy was external beam radiotherapy (*n* = 366, 93.1%), followed by radioactive implants (*n* = 4, 1.0%) and radioisotopes (*n* = 2, 0.5%). The remaining cases were not further specified. The majority of patients (*n* = 774, 45.7%) had dedifferentiated liposarcoma, followed by well-differentiated liposarcoma (*n* = 556, 33.5%), liposarcoma not otherwise specified (*n* = 208, 12.3%), and myxoid liposarcoma (*n* = 73, 4.3%). The remaining cases were classified as other types of liposarcoma, including myxoid liposarcoma (*n* = 73, 4.3%), pleomorphic liposarcoma (*n* = 36, 2.1%), mixed liposarcoma (*n* = 27, 1.6%), and round cell liposarcoma (*n* = 8, 0.5%).

Table 1 displays the demographic, clinical, and pathological characteristics. In unadjusted analyses, there were no significant differences in the proportion of female patients, age, and race between the groups. Patients who received radiotherapy were more often married, had a higher rate of tumors sized between 10 and 20 cm, unknown tumor grading, and chemotherapy application. The results after propensity-score matching are presented in Table 1, confirming the balanced characteristics after matching.

**Table 1:** Demographic, clinical, and pathologic characteristics of patients with localized retroperitoneal liposarcoma who underwent surgery between 2004 and 2020. Patients were divided according to the use of perioperative radiotherapy

	Before PSM			After PSM (2:1)			
	No/unknown [*n* = 1299]	Radiotherapy [*n* = 393]	*p*-Value^a^	No/unknown [*n* = 701]	Radiotherapy [*n* = 384]	*p*-Value^a^	SMD
Sex			0.075			0.59	
Female	595 (45.8)	160 (40.7)		302 (43.1)	159 (41.4)		−0.021
Male	704 (54.2)	233 (59.3)		399 (56.9)	225 (58.6)		0.021
Age, years [mean (SD)]	62.6 (12.9)	61.7 (12.0)	0.25	62.6 (12.5)	62.1 (11.8)	0.48	−0,047
Race			0.078			0.20	NA
White	1093 (84.1)	332 (84.5)		585 (83.5)	324 (84.4)		
Black	76 (5.9)	13 (3.3)		38 (5.4)	12 (3.1)		
Other/unknown	130 (10.0)	48 (12.2)		78 (11.1)	48 (12.5)		
Marital status			0.013			0.023	NA
Married	815 (62.7)	274 (69.7)		445 (63.5)	268 (69.8)		
Single/separated/divorced/ Widowed	417 (32.1)	109 (27.7)		216 (30.8)	106 (27.6)		
Other/unknown	67 (5.2)	10 (2.5)		40 (5.7)	10 (2.6)		
Tumor size, cm			<0.001			0.50	
≤10	221 (17.0)	74 (18.8)		111 (15.8)	74 (19.3)		0.060
10–20	396 (30.5)	160 (40.7)		288 (41.1)	155 (40.4)		−0,024
>20	610 (47.0)	145 (36.9)		271 (38.7)	141 (36.7)		−0.016
Unknown	72 (5.5)	14 (3.6)		31 (4.4)	14 (3.6)		−0.021
Grading			<0.001			0.046	
I/II – well/intermediate differentiated	723 (55.7)	145 (36.9)		293 (41.8)	145 (37.8)		−0.008
III/IV – poorly/ undifferentiated	449 (34.6)	154 (39.2)		295 (42.1)	154 (40.1)		0.024
Unknown	127 (9.8)	94 (23.9)		113 (16.1)	85 (22.1)		−0.018
Chemotherapy			<0.001			0.002	NA
No/unknown	1192 (91.8)	330 (84.0)		636 (90.7)	324 (84.4)		
Yes	107 (8.2)	63 (16.0)		65 (9.3)	60 (15.6)		

Data are expressed as *n* (%) unless otherwise specified

*SMD* standardized mean difference, *SD* standard deviation, *NA* not available, *PSM* propensity-score matching

^a^*P*-values were derived using Pearson’s Chi-square test for categorical variables and t-test for continuous variables

### Survival Analysis

Overall, 24.3% (*n* = 316) of patients who did not receive radiotherapy died of RPLS, while in the group of patients who received radiotherapy, the rate of patients who died of RPLS was 28.1% (*n* = 111). Median survival was 45 months in the group of patients without radiotherapy and 46 months in the group of patients who had radiotherapy.

**Fig. 2:**
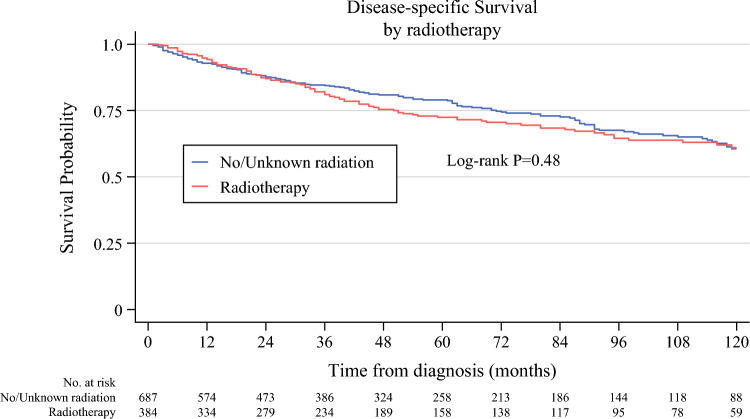
Disease-specific survival of patients with localized retroperitoneal liposarcoma who underwent surgery between 2004 and 2020 after propensity score matching

**Fig. 3:**
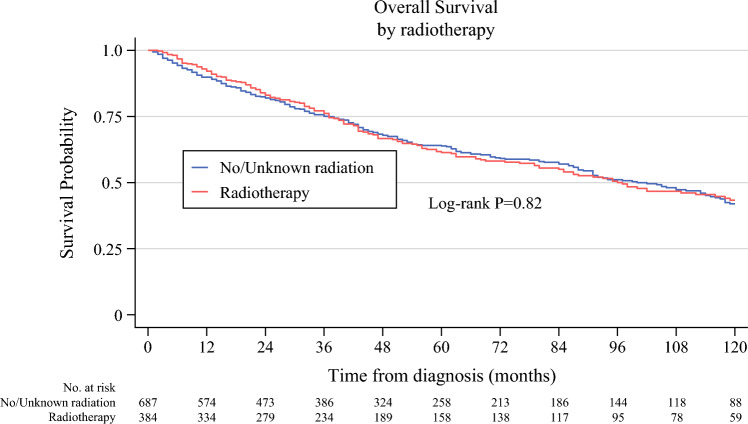
Overall survival of patients with localized retroperitoneal liposarcoma who underwent surgery between 2004 and 2020 after propensity score matching

**Fig. 4:**
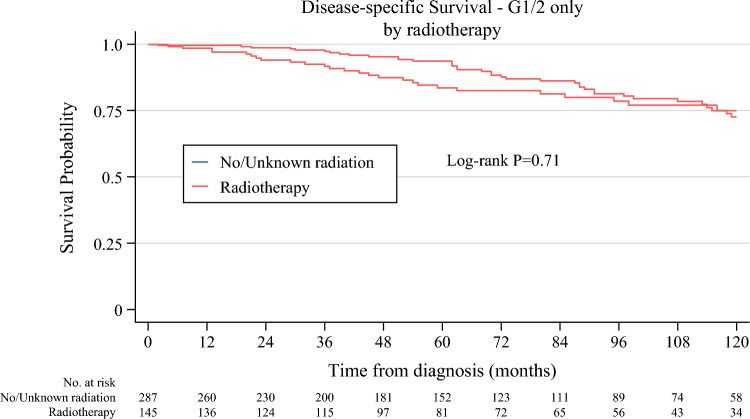
Subgroup analysis of disease-specific survival in patients with G1 and G2 localized retroperitoneal liposarcoma who underwent surgery after propensity score matching

Kaplan–Meier analysis was performed before and after propensity score matching. Kaplan–Meier curves prior to propensity-score matching demonstrated no statistically significant difference in DSS between the two groups (electronic supplementary material [ESM] Fig. 1). After propensity-score matching, the 5- and 10-year DSS rates were 72.5% and 61.0% in the group of patients who received radiotherapy, and 79.0% and 60.6% in the group of patients who did not receive radiotherapy, respectively (log-rank *p* = 0.48) (Fig. 2). Five-year overall survival was 61.3% with radiotherapy and 63.8% without radiotherapy (log-rank *p* = 0.82) (Fig. 3). Furthermore, multivariable adjusted Cox regression analysis after propensity score matching did not demonstrate an improvement of DSS after perioperative radiotherapy (adjusted hazard ratio [aHR] 1.04, 95% confidence interval [CI] 0.81–1.32) [Table 2]. Patient age >80 years (aHR 1.71, CI 1.03–2.86), tumor size between 10 and 20 cm, and tumor size >20 cm compared with tumor size ≤10 cm (aHR 1.90, CI 1.22–2.96, and aHR 2.76, CI 1.78–4.28, respectively), and tumor grade G3 versus G1/2 (aHR 3.28, CI 2.47–4.37) were associated with an increased risk of death from RPLS. To address the potential for selection bias, subgroup analysis was performed, with patients stratified into grade 1/2 and grade 3 groups. Subgroup analysis demonstrated no differences in DSS (Fig. 4).

**Table 2:** Multivariable-adjusted Cox proportional hazards regression of death from localized retroperitoneal liposarcoma in patients undergoing surgery between 2004 and 2020 after propensity score matching

Variable	aHR^a,b^ [CI]	*p*-Value
Radiotherapy		
Any radiotherapy	1.04 [0.81–1.32]	0.77
Sex		
Male	1.19 [0.93–1.52]	0.17
Age, years		
60–79	1.18 [0.92–1.52]	0.20
≥80	1.72 [1.03–2.86]	0.04
Tumor size, cm		
10–20	1.90 [1.22–2.96]	0.01
>20	2.76 [1.78–4.28]	<0.01
Unknown	3.23 [1.65–6.35]	<0.01
Grade		
III/IV – poorly/undifferentiated	3.28 [2.47–4.37]	<0.01
Unknown	2.15 [1.46–3.16]	<0.01

^a^Multivariate Cox regression model adjusted for application of radiotherapy, sex, age, tumor size and grade

^b^Reference categories: No/unknown radiotherapy, female sex, patient age <60 years, tumor size <10 cm, grade I/II

*aHR* adjusted hazard ratio, *CI* 95% confidence interval

After propensity-score matching and multivariable adjustment, logistic regression demonstrated that radiotherapy was more likely to be administered if tumor grading was unknown (adjusted odds ratio [aOR] 1.49, 95% CI 1.04–2.14) (Table 3). Radiotherapy was also associated with systemic treatment (aOR 1.75, CI 1.18–2.59).

**Table 3:** Multivariable-adjusted odds of radiotherapy in patients with localized retroperitoneal liposarcoma undergoing surgery between 2004 and 2020 after propensity score matching

Variable	aOR^a,b^ [CI]	*p*-Value
Sex		
Male	1.04 [0.80–1.35]	0.79
Age, years		
60–79	0.94 [0.72–1.23]	0.65
≥80	0.82 [0.46–1.45]	0.50
Race		
Black	0.59 [0.30–1.16]	0.13
Other/unknown	1.20 [0.81–1.78]	0.36
Marital status		
Single/separated/divorced/widowed	0.80 [0.60–1.07]	0.14
Other/unknown	0.40 [0.19–0.82]	0.01
Tumor size, cm		
10–20	0.80 [0.56–1.15]	0.23
>20	0.81 [0.56–1.17]	0.26
Unknown	0.69 [0.33–1.42]	0.31
Grade		
III/IV–poorly/undifferentiated	0.98 [0.73–1.30]	0.88
Unknown	1.49 [1.04–2.14]	0.03
Chemotherapy		
Yes	1.75 [1.18–2.59]	0.01

^a^Multivariable logistic regression model adjusted for application of radiotherapy, sex, age, race, marital status, tumor size, grade, and application of chemotherapy

^b^Reference categories: no radiotherapy, female sex, age <60 years, White race, married marital status, tumor size <10 cm, grade I/II, no chemotherapy

*aOR* adjusted odds ratio, *CI* 95% confidence interval

## DISCUSSION

This study provides an updated analysis of patients with localized RPLS, the group of patients that has been shown to possibly benefit the most from perioperative radiotherapy. The current population-based cohort study found no benefit of perioperative radiotherapy regarding disease-specific and overall survival in patients with localized RPLS who underwent surgery. The main predictors of death of RPLS were older patient age, larger tumor size, and advanced tumor grading.

The STRASS trial was the largest prospective, randomized trial performed that assessed the effect of preoperative radiotherapy on abdominal RFS in localized RPS of various histologies.^6^ No benefit of preoperative radiotherapy on 3-year abdominal RFS has been shown; however, in subgroup analyses, there was an absolute benefit of 10% in abdominal RFS for patients with RPLS, which was not statistically significant (HR 0.62, 95% CI 0.38–1.02).^6^ The consecutive pooled analysis of STRASS trial data and off-trial data (STREXIT) revealed a beneficial effect of preoperative radiotherapy on abdominal RFS for well-differentiated and G1–2 dedifferentiated liposarcoma (HR 0.63, 95% CI 0.40–0.97), without affecting the abdominal RFS of G3 dedifferentiated liposarcoma.^10^ The authors thus concluded that preoperative radiotherapy should be supported in well-differentiated and G1–2 dedifferentiated RPLS but not in G3 dedifferentiated liposarcoma. Preoperative radiotherapy did not influence overall or distant metastasis-free survival according to the results of the STRASS or STREXIT trials. Recruitment for the STRASS trial was a significant challenge due to the rarity of RPS. The involvement of 31 centers over a 5-year period was essential to recruit a total of 266 patients. The STRASS trial encompassed not only RPLS but also other forms of RPS. Given this, it seems to be impractical to conduct a prospective randomized trial that exclusively includes patients with RPLS. Consequently, the current research question can only be addressed in population-based studies to date. The findings of the STRASS and STREXIT trials were further corroborated by a recently published propensity score-matched analysis of the NCDB, which included patients with RPLS undergoing surgery with and without perioperative external beam radiotherapy during 2004 and 2017. No association between perioperative radiotherapy and overall survival could be demonstrated for either well-differentiated or dedifferentiated RPLS.^11^ In contrast, previous population-based studies involving a more heterogeneous group of RPSs have demonstrated a survival benefit following perioperative radiotherapy. Nussbaum et al. conducted a retrospective propensity score-matched analysis of the NCDB including 9068 adult patients diagnosed with RPS between 2003 and 2011. These patients underwent either perioperative radiotherapy and surgery or surgery alone.^7^ A median overall survival of 89 months (95% CI 79–100 months) was observed in patients who underwent radiotherapy and surgery, in comparison with a median overall survival of 64 months (95% CI 59–69 months) in patients who underwent surgery alone. A retrospective analysis of 1266 patients with surgically treated localized RPS based on the SEER database from 2004 to 2014 was conducted by Nazzani et al. that demonstrated a reduction in disease-specific mortality (HR 0.73, CI 0.55–0.96) in patients undergoing radiotherapy.^2^ In contrast to the current study, the analysis conducted by Nazzani et al. included a range of RPSs, rather than exclusively focusing on RPLS. A more homogeneous group consisting only of localized RPLS was examined retrospectively by the Transatlantic Retroperitoneal Sarcoma Working Group. In this study population consisting of 607 patients, unadjusted analysis showed an improvement in local recurrence rate in favor of perioperative radiotherapy. However, this difference vanished after adjustment for potential bias with propensity-score matching.^19^ The current study, which comprises an updated population-based cohort of patients with RPLSs, did not identify any impact of perioperative radiotherapy on DSS. The DSS measure focuses on quantifying the time from diagnosis or commencement of therapy until death caused by the underlying disease, excluding deaths resulting from unrelated causes. The SEER database employs algorithms to analyze the underlying cause of death as indicated on death certificates, identifying a single disease-specific cause. In this context, the SEER database is one of a few databases that provides DSS as an endpoint. DSS provides a more precise estimation of the underlying cause of death than overall survival, which may be confounded by the presence of other competing causes of death. Additionally, DSS can assist in determining whether a treatment improves survival outcomes by controlling the disease, independent of other factors. Furthermore, DSS can be utilized to distinguish between individuals living with a controlled disease and those who have been cured or have developed different health conditions.

A report from the Transatlantic Retroperitoneal Sarcoma Working Group emphasized that local recurrence of RPS is associated with a lower likelihood of survival.^20^ The contrast between the improved local control in the STRASS study and the lack of survival benefit in our study could be due to several factors. Treatment techniques and strategies for treating local recurrence have likely evolved in recent years. In addition, the time between local recurrence and death can be very long, especially in well-differentiated RPLSs.^21^ It is therefore possible that some deaths were not recorded during follow-up. Additionally, the proportion of patients who underwent neoadjuvant radiotherapy in the present study was 35%. In contrast, the STRASS trial exclusively included patients who underwent neoadjuvant radiotherapy; however, excluding patients who received postoperative radiotherapy from this analysis would have made statistical evaluation difficult and less reliable due to the limited number of cases. Given that the SEER is one of the largest databases available that captures DSS, it remains a challenge to address this issue in a more targeted way.

The primary predictors of survival of patients with localized RPLS in our study were advanced patient age, larger tumor size, and advanced tumor grading. This is consistent with the findings of previous studies that have identified pathological grading as a significant predictor of RFS and DSS.^22–25^ A retrospective, single-center study by Zhao et al. found that the median overall survival in low- and high-grade RPLS following surgical resection was 105 months and 34 months, respectively.^25^ The mortality rates observed in the present study for different grades of RPLS are consistent with those previously reported in the literature; however, radiotherapy had no effect on DSS in a subgroup analysis of G1–2 RPLS.

This study has several limitations. First, it should be noted that the SEER is a retrospective database, which is subject to limitations inherent to retrospective study designs. In general, retrospective studies can only describe associations and cannot determine causality. Furthermore, they can be subject to selection bias and coding errors. The findings of this retrospective study are therefore not directly comparable with the randomized STRASS trial. On the other hand, it can be assumed that a high level of generalizability is present, given the population-based nature of the registry. The SEER database was not originally designed to determine specific treatment outcomes. Nevertheless, population-based databases, such as the SEER database, are of critical importance in the assessment of rare diseases, including RPLS. A highly relevant prognostic factor is the completeness of resection.^25,26^ It was not possible to include positive margins or R status in our analysis, as this is not covered by the SEER database. Additionally, the SEER database lacks information regarding comorbidity status, performance status, facility type, and multifocality and molecular data. Therefore, no confounding data related to comorbidities could be included from this dataset. Moreover, the SEER database lacks detailed information regarding treatment modalities, radiotherapy dose, radiation field, completion of radiotherapy, and the number of fractions. It is plausible that these variables may have impacted the analysis. A further limitation of the SEER database is that it lacks information on treatments received outside the designated catchment area. Consequently, some treatments that were administered outside the hospitals may be missed. In contrast, the surgical information is anticipated to be largely comprehensive. The SEER database only reports data on radiotherapy administered as a first-course treatment. It is possible that patients may have received additional therapy during the course of their treatment that has not been recorded in the database.^27^ The SEER database lacks information regarding the rationale behind the choice of treatment modality. It is thus unclear whether patients who did not undergo treatment were presented with therapeutic options but ultimately chose to decline them. Chemotherapy may influence the outcomes of patients with malignant diseases; however, there are reasons for not including chemotherapy in the propensity-score matching. The indication for chemotherapy is unclear. It can be used as a surrogate for potentially more advanced disease. Additionally, since there are great variations of treatment regimens, especially in RPLS, it is likely that there are facilities that use chemotherapy as a standard therapy for all patients regardless of disease stage. Moreover, according to a comparison of the SEER database with Medicare data, the chemotherapy variable has a sensitivity of 68%.^28^ Patients that are coded as not having received chemotherapy may have received therapy in a later course of disease, in another facility or in an outpatient setting that was not captured by the SEER database. The aforementioned comparison of the SEER database with Medicare data showed a high sensitivity of 80% for the SEER radiotherapy data. The overall positive predictive value for all treatments exceeded 85%.^28^ It is possible that a small number of patients may have undergone radiotherapy that was not included in the SEER dataset. It has to be noted that the SEER database does not distinguish between ‘no’ and ‘unknown’ radiotherapy status. Certain limitations of DSS must be considered. Cancer registries such as the SEER rely on algorithms to interpret causes of death from death certificates and assign a single, disease-specific, underlying cause of death; however, death certificates may be inaccurately classified or may be incorrect. Additionally, the SEER database lacks comprehensive data on comorbidities, complicating the determination of specific causes of death in some cases. To reduce misclassification, the algorithm developed by Howlader et al. in 2010 was employed.^29^

Nevertheless, the SEER-17 database encompasses 26.8% of the US population, thereby allowing for the assumption of a high degree of generalizability. Only population-based registries that comprehensively collect data regarding survival outcomes can validate the results of RCTs, thereby confirming their relevance and applicability to real-world scenarios. In light of the conclusions of the STRASS and STREXIT studies with reduced recurrence rates after preoperative radiotherapy, it is possible to infer that neoadjuvant radiotherapy may be evaluated in selected cases of localized RPLS, such as those involving borderline resectable or unresectable tumors. Prerequisite is a sufficient target volume delineation and treatment planning. A retrospective analysis of the STRASS trial radiotherapy technique revealed a significant improved abdominal RFS in the subgroup of patients passing the quality assurance criteria;^30^ however, our data do not support the routine use of radiotherapy in patients with resectable, localized RPLS.

## CONCLUSION

The administration of perioperative radiotherapy did not improve DSS in patients undergoing surgery for localized RPLS in this population-based study; therefore, the use of perioperative radiotherapy in these patients may be questioned. However, the findings should be interpreted with caution due to the inherent limitations of the SEER database.

## Supplementary Information

Below is the link to the electronic supplementary material.

Fig. 1 Disease-specific survival of patients with localized retroperitoneal liposarcoma who underwent surgery between 2004 and 2020 before propensity score matching.
Supplementary file1 (JPG 443 KB)
