# Supplementary figures and images for: The Impact of Perioperative Radiotherapy on Disease-Specific Survival in Patients with Localized Retroperitoneal Liposarcoma: A Population-Based Propensity-Score Matched Analysis
Source: Ann Surg Oncol. 2024 Dec 16;32(3):1541–9. doi: 10.1245/s10434-024-16703-w (PMC11811447; doi:10.1245/s10434-024-16703-w)

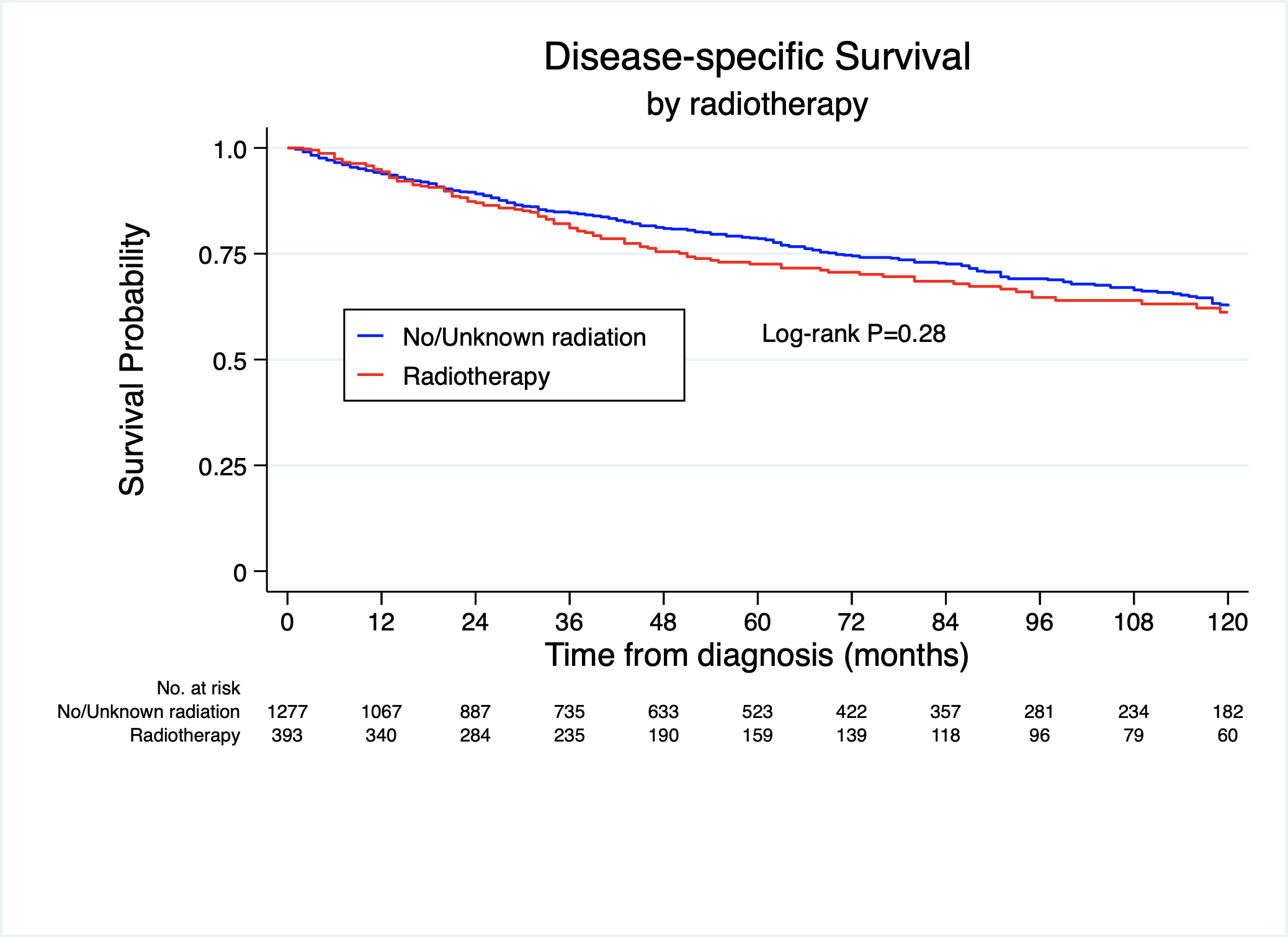

Supplement: Supplementary file 1 — Disease-specific survival of patients with localized retroperitoneal liposarcoma who underwent surgery between 2004 and 2020 before propensity score matching. Supplementary file1 (JPG 443 KB) [file 10434_2024_16703_MOESM1_ESM.jpg]
